# Central Nervous System Inflammatory Aggregates in the Theiler's Virus Model of Progressive Multiple Sclerosis

**DOI:** 10.3389/fimmu.2019.01821

**Published:** 2019-08-02

**Authors:** Krista D. DiSano, Darlene B. Royce, Francesca Gilli, Andrew R. Pachner

**Affiliations:** Department of Neurology, Dartmouth Hitchcock Medical Center and Geisel School of Medicine, Lebanon, NH, United States

**Keywords:** multiple sclerois, ectopic lymphoid follicles, B cells, TMEV-IDD, neuroinflammation

## Abstract

Persistent central nervous system (CNS) inflammation, as seen in chronic infections or inflammatory demyelinating diseases such as Multiple Sclerosis (MS), results in the accumulation of various B cell subsets in the CNS, including naïve, activated, memory B cells (Bmem), and antibody secreting cells (ASC). However, factors driving heterogeneous B cell subset accumulation and antibody (Ab) production in the CNS compartment, including the contribution of ectopic lymphoid follicles (ELF), during chronic CNS inflammation remain unclear and is a major gap in our understanding of neuroinflammation. We sought to address this gap using the Theiler's murine encephalomyelitis virus-induced demyelinating disease (TMEV-IDD) model of progressive MS. In this model, injection of the virus into susceptible mouse strains results in a persistent infection associated with demyelination and progressive disability. During chronic infection, the predominant B cell phenotypes accumulating in the CNS were isotype-switched B cells, including Bmem and ASC with naïve/early activated and transitional B cells present at low frequencies. B cell accumulation in the CNS during chronic TMEV-IDD coincided with intrathecal Ab synthesis in the cerebrospinal fluid (CSF). Mature and isotype-switched B cells predominately localized to the meninges and perivascular space, with IgG isotype-switched B cells frequently accumulating in the parenchymal space. Both mature and isotype-switched B cells and T cells occupied meningeal and perivascular spaces, with minimal evidence for spatial organization typical of ELF mimicking secondary lymphoid organs (SLO). Moreover, immunohistological analysis of immune cell aggregates revealed a lack of SLO-like ELF features, such as cell proliferation, cell death, and germinal center B cell markers. Nonetheless, flow cytometric assessment of B cells within the CNS showed enhanced expression of activation markers, including moderate upregulation of GL7 and expression of the costimulatory molecule CD80. B cell-related chemokines and trophic factors, including APRIL, BAFF, CXCL9, CXCL10, CCL19, and CXCL13, were elevated in the CNS. These results indicate that localization of heterogeneous B cell populations, including activated and isotype-switched B cell phenotypes, to the CNS and intrathecal Ab (ItAb) synthesis can occur independently of SLO-like follicles during chronic inflammatory demyelinating disease.

## Introduction

The presence of antibody (Ab), antibody-secreting cells (ASC), and multiple other B cell subsets in the CNS compartment are hallmarks of chronic inflammatory processes during persistent infection or demyelinating diseases. In the inflammatory demyelinating disease Multiple Sclerosis (MS), CNS-infiltrating B cells are associated with the presence of oligoclonal IgG bands in the cerebrospinal fluid (CSF), a diagnostic hallmark in 95% of patients resulting from intrathecal antibody (ItAb) production in the CNS compartment (1–3). The exact role of B cells and Ab in MS remains controversial, but nevertheless multiple B cell phenotypes are implicated in MS pathogenesis. IgG and complement deposition are frequently observed in active demyelinating lesions in MS (4–7), indicating local Ab and complement-mediated mechanisms may contribute to demyelination. The success of B cell depletion therapies (BCDT) targeting CD20^+^ B cells, including mature B cells to plasmablasts, have further implicated B cells in MS pathogenesis, as BCDT reduces the formation of new inflammatory lesions and relapses in relapsing-remitting MS (RRMS) patients and time to confirmed disease progression in young, inflammatory primary progressive MS (PPMS) patients with minimal effects on ItAb (8–12). Collectively, these findings implicate a critical, yet unclear role for multiple B cell phenotypes in MS pathogenesis. However, the mechanisms underlying the recruitment and survival of diverse B cell populations in the CNS as well as factors responsible for fostering Ab production within the CNS compartment in MS and other chronic neuroinflammatory diseases are still largely undetermined.

The exact mechanisms regulating B cell accumulation and persistence in tissues outside of secondary lymphoid organs (SLO) remain unclear. Classically, infiltration of immune cells into non-lymphoid tissue sites, including the CNS, was seen as a random and diffuse process. However, during chronic inflammation lymphoid neogenesis may occur in certain conditions whereby tissue-infiltrating immune cells form highly organized structures resembling SLOs. These structures, known as tertiary lymphoid organs or ectopic lymphoid follicles (ELF), are locally inducible within chronically inflamed sites and contain the organizational, cellular, and molecular features found in SLO essential for propagating immune cell activation and antigen-driven selection, ultimately sustaining tissue-specific immune responses (13–16). Furthermore, similar to SLO, ELF contain B cell-rich infiltrates and are implicated in promoting focal B cell organization to aid activation, affinity maturation, differentiation, class-switch recombination, and local Ab production (13, 16, 17). ELF can develop in nearly every organ in the body during infection, autoimmunity, and tumorigenesis, although the degree to which ELF recapitulate lymphoid tissue-like organization varies (13, 16, 18). These structures can provide a permissive environment for cellular and humoral immune responses independent of primary or secondary lymphoid tissue.

ELFs are particularly prominent during chronic inflammation, yet mouse models of neuroinflammation examining CNS compartmentalized immune responses, including ELF formation, have focused on acute rather than chronic disease. Studies in murine models of CNS demyelinating diseases including mouse hepatitis virus (MHV) (19–23), Sindbis virus infection (24, 25), and experimental autoimmune encephalomyelitis (EAE) (18, 26–29) have highlighted the ability for the CNS to serve as a niche for persistent B cell accumulation. Critical B cell chemokines and trophic factors are constitutively expressed or upregulated during neuroinflammation, including trafficking chemokines, CXCL9/10 and CCL19/21, SLO-organizing chemokines CXCL13 and CXCL12, and survival factors and differentiation factors, including BAFF, APRIL, and IL-21 (19, 20, 22, 26, 27, 30–34). Both infiltrating immune cells and CNS-resident cells, including astrocytes, microglia, and endothelial cells, produce B cell-related chemoattractants and trophic factors in the CNS (19). However, the relative contribution of ELF in fostering CNS compartmentalized B cell responses during acute models of MS remains controversial. In adoptive transfer EAE models, ELFs have been noted within the meninges and express some characteristic lymphoid factors including IL-17, LTα, and chemokines involved in leukocyte homing to and within the lymph nodes such as CXCL13, CXCL9, and CCL19 (28, 35–38).

Conversely, MHV infection, as well as spontaneous B cell-dependent 2D2 IgH^MOG^ MOG EAE, have noted the presence of multiple B cell differentiation phenotypes, including activated and proliferating B cells and lymphoid chemokines with minimal evidence for SLO-like ELF (23, 29). Collectively, studies in viral and autoimmune models emulating the acute phase of MS support the notion that prolonged B cell responses within the CNS can occur irrespective of ELF formation. Nonetheless, ELFs are classically induced during chronic inflammation and therefore may play a more critical role in chronic and progressive phases of the disease. Studies documenting meningeal inflammatory aggregates resembling some features of ELF were found primarily in secondary progressive MS (SPMS) post-mortem brain tissue (36, 37, 39), yet, mouse models of chronic and progressive demyelinating disease course have not evaluated contributions of ELF in promoting CNS compartmentalized immune responses. Thus, the contribution of ELF in promoting persistent B cell activity in the CNS during chronic and progressive phases of MS requires further evaluation.

The pathology of progressive MS is complex and recapitulating all facets of progressive disease in an individual mouse model has proven to be challenging. Nonetheless, Theiler's murine encephalomyelitis virus (TMEV)-induced demyelinating disease (TMEV-IDD) has been established as an accepted model of chronic MS, especially its progressive forms. Chronic TMEV infection of the CNS in susceptible strains of mice produces a progressive clinical disease course exemplified by accrued disability, including spastic paralysis. The model further parallels several hallmarks of progressive MS exhibiting both spinal cord and brain pathology, including demyelination, remyelination, atrophy, neuronal death, axonal injury, and persistent CNS inflammation (40–43). Furthermore, several studies document ItAb synthesis in the CNS during TMEV-IDD, similar to MS (44, 45). The phenotype of accumulating B cells and the role of ELF in supporting prolonged CNS compartmentalized inflammation and Ab production in TMEV-IDD has yet to be determined. Thus far, persistent accumulation of T cells and B cells (46) in the CNS during chronic TMEV-IDD and elevations in CSF Ab despite restored blood-brain barrier (BBB) integrity (46, 47) suggest CNS compartmentalized cellular and humoral immune responses may be fostered during chronic TMEV-IDD.

The present study sought to elucidate B cell phenotypes accumulating in the CNS, and the relative contribution of CNS compartmentalized immune responses, including SLO-like ELF formation, in sustaining B cell accumulation and Ab production in the CNS in TMEV-IDD. Overall, our results demonstrate that ongoing B cell responses and ItAb synthesis in the CNS during chronic TMEV occur independent of SLO-like ELF formation. Our findings broaden our view of the diverse factors involved in supporting CNS compartmentalized B cell responses during CNS inflammatory diseases, a crucial finding for identifying future therapeutic interventions targeting inflammatory aggregates.

## Methods

### Mice and Infection

Four to five-week-old female SJL mice were purchased from Jackson Laboratories (Bar Harbor, ME) and were intracranially infected with 10 ×10^6^ plaque forming units (PFU) of TMEV (BeAn strain) in 30 μl final volume. All mice were housed under pathogen-free conditions at an accredited facility at Dartmouth College. The control sham-treated group received an intracranial injection with 30 μl of saline solution and were age-matched to TMEV-IDD mice for all experiments. All procedures were conducted under protocols approved by the Institutional Animal Care and Use Committee.

### Sample Isolation

Whole blood, cervical lymph nodes (CLN), spinal cord, and CSF were obtained from each mouse at necropsy (*n* = 32 TMEV-IDD and *n* = 12 sham from 4 independent experiments). Blood (average 500 μl) was collected by intracardiac puncture and serum was isolated and stored at −80°C. CLN were either paraffin-embedded for immunofluorescence studies (*n* = 11 TMEV-IDD; *n* = 3 sham) or processed for flow cytometry (*n* = 11 TMEV-IDD; *n* = 3 sham). Spinal cords were divided, with 1/3 of tissue snap frozen and stored at −80°C for gene expression analyses and 2/3 of tissue either left in the spinal column, with vertebrae intact for paraffin-embedding (*n* = 8 TMEV-IDD; *n* = 6 sham) or flushed from the spinal column to process for flow cytometry (*n* = 24 TMEV-IDD; *n* = 6) as noted below. CSF was collected by cisternal tap as previously described (48). Briefly, the meninges overlaying the cisterna magna were exposed, the surrounding area was gently cleaned to remove any contaminating blood, and a 30 gauge needle was used to puncture the arachnoid membrane. CSF was collected using a glass capillary tube (average 8–10 μl), centrifuged to remove cells, diluted 1:3 in PBS, and stored at −80°C.

### RNA Preparation and Real-Time Quantitative Reverse Transcription (RT-PCR)

RNA was extracted from spinal cords using TRIzol (Invitrogen, Foster City, CA). RNA was reverse transcribed using the qScript cDNA Supermix kit (Quanta-Biosciences, Gaithersburg, MD). cDNA was then utilized as the template for real-time RT-PCR based on the 5′ nuclease assay, using the PerfeCTa qPCR FastMix II ROX (Quanta-Biosciences, Gaithersburg, MD). Custom primers and probes were used to detect TMEV mRNA (45), and TaqMan real-time PCR assays (Life Technologies, Grand Island, NY) were used as the primers and probes for all other target genes, including mouse glyceraldehyde phosphate dehydrogenase (GAPDH), the reference gene. TMEV mRNA was assessed by absolute quantification using a standard curve of TMEV plasmids amplified at known concentrations. For the present study, only TMEV positive mice were included in our data analysis. All other targets were analyzed as relative mRNA expression levels calculated by using both the 2^−Δ*Ct*^ method where ΔCt = Ct_target_−*Ct*_GAPDH_, and the 2^−ΔΔ*Ct*^ methods where ΔΔCt = Δ*Ct*_TMEV−IDD_−Δ*Ct*_Sham_ (49).

### Luminex® Bead-Based Multiplex Assay

Immunoglobulins (Ig) and cytokine levels in the CSF and serum were measured using Luminex® Bead-based Multiplex Assay. The MilliPlex MAP Mouse Immunoglobulin Isotyping Magnetic Bead Panel (EMD Millipore, Burlington, MA) was used to quantify levels of IgA, IgG1, IgG2a, IgG2b, IgG3, and IgM. Likewise, the Bio-Plex Pro Mouse Chemokine Panel 33-plex assay (Bio-Rad, Cambridge, MA) was used to quantify 33 chemokines, including B cell-related chemokines CXCL13, CCL19, CXCL10, IL-6, and CXCL12. Mouse CXCL9 and IL-21 were measured using Luminex® singleplex assays available from Bio-Rad. In order to compare intrathecal production of Igs and chemokines, CSF analytes were normalized to serum analytes by calculating a CSF/serum ratio, which accounts for individual variability in serum concentrations (50, 51).

### Cell Isolation and Flow Cytometry

For B cell analysis via flow cytometry, CLN cells or spinal cord-derived mononuclear cells were isolated from individual TMEV infected or sham mice as previously described (23). Briefly, spinal cords were minced and digested in RPMI supplemented with 10% fetal calf serum (FCS), collagenase type I (1 mg/ml; Worthington Biochemical Corporation, Lakewood, NJ) and DNase I (100 U/ml) (Worthington Biochemical Corporation, Lakewood, NJ). Incubation was followed by the addition of 0.1M EDTA to terminate collagenase activity. Cell pellets were resuspended in RPMI medium, adjusted to 30% Percoll (Pharmacia, Piscataway, NJ), and underlaid with 1 ml of 70% Percoll. Cells were recovered from the 30/70% Percoll interface. Prior to staining, cells were incubated with fixable viability stain 780 APC-Cy7 (BD Biosciences, San Jose, CA). Cells were then incubated with FACS buffer supplemented with 1% mouse serum and rat anti-mouse FcγIII/II mAb (2.4G2; BD Bioscience, San Jose, CA) to prevent non-specific staining. Expression of B cell phenotype and activation surface markers was assessed by staining CLN or CNS cells with Ab specific for CD45 (30-F11; PerCP-Cy5.5), CD19 (1D3; PE-CF594), GL7 (FITC), IgD (11-26; APC), IgM (eB131-15F9; PE), CD138 (281-2; PE), IgG2a/2b (R2-40; FITC) IgG1 (A85-1; FITC), MHCII IA^q^ (KH116; BV421), CD80 (16-10A1; PE) (BD Biosciences, San Jose, CA) (Table 1). Cells were analyzed using a Beckmann Coulter Gallios flow cytometer (BD Biosciences, San Jose, CA) and FlowJo (version 9.7.6) software (Tree Star, Ashland, OR). Voltages for the cytometer's detectors were set based on B cell surface marker expression on B cells isolated from CLN and the same voltages were utilized for analyzing CNS-infiltrating B cells. Cell numbers were calculated based on live cell yields and percentages of gated live cells. Dead cells were excluded using fixable viability stain and comprised <10% of isolated cells. Doublets were excluded based on FSC-Area and FSC-Height.

**Table 1:** Expression of phenotypic and activation markers on B cells, including naïve, transitional (Tran), activated (Activ), isotype-unswitched (Iso-unswitched), and isotype-switched (Iso-switched) subpopulations (52–60).

**Marker**	**Naïve**	**Tran**	**Activ**	**GC**	**Iso-unswitched**			**Iso-switched**		
					**Bmem**	**PB**	**PC**	**Bmem**	**PB**	**PC**
B220	+	+	+	+	+	hi	lo	+	hi	lo
CD19	+	+	+	+	+	hi	lo	+	hi	lo
IgD	+	int	-	-	-	-	-	-	-	-
IgM	+	+	+	+	+	+	+	-	-	-
IgG	-	-	-	-	-	-	-	+	+	+
CD138	-	-	-	-	-	+	+	-	+	+
GL7	-	-	lo	hi	-	-	-	-	-	-
CD80	-	lo	hi/+	+	+	-	-	+	-	-
MHCII	+	+	+	+	+	+	lo	+	+	lo

### Immunohistochemistry

Spinal cords and CLN from PBS-perfused, TMEV-IDD and sham mice were fixed in 10% neutral buffered formalin for 24 h. To retain the meninges, the vertebral column was isolated, trimmed, and spinal cords with intact vertebrae were segmented into cervical, upper thoracic, and lower thoracic regions and embedded in paraffin. Tissue blocks were then surface decalcified using Nitrical nitric acid bone decalcifier (StatLab medical products, McKinney, TX) for 5-10 min, washed, and cut in sections of 4 μm thickness. Deparaffinization and rehydration steps were performed using two xylene 10-min washes followed by two 10-min 100% ethanol washes, and sequential 5-min washes of 95% ethanol, 70% ethanol, and 50% ethanol. Following PBS washes, antigen retrieval was performed at 95°C for 20 min in Tris-EDTA 0.1% Tween-20 buffer followed by cooling. Sections were washed in PBS and blocked with 5% bovine serum albumin and 10% goat serum for 1 h. After blocking, spinal cord sections were incubated with primary antibodies (Table 2). B220 and CD21/35 primary Abs were detected using secondary Ab Alexa Fluor 594 goat anti-rat IgG (Abcam), and caspase-3, Ki-67, CD3, and laminin primary Abs were detected using secondary Ab Alexa Flour 488 goat anti-rabbit IgG (Life Technologies, Grand Island, NY). Sections were mounted with Vectashield Hardset reagent with 4′,6-diamidino-2-phenylindole (DAPI) (Vector Labs, Burlingame, Ca) and examined using a Zeiss LSM 800 confocal microscope with Airyscan (Zeiss, Oberkochen, Germany). Z-series images were collected every 0.2 μm covering a tissue depth of 2-3 μm. Projected images were compiled using Image J software (NIH, http://rsbweb.nih.gov/ij) supplemented with the FIJI plugin set (https://imagej.net/Fiji). Images were assembled for publication and scale bars added using Adobe Photoshop 7.0 software (Adobe Systems, San Jose CA).

**Table 2:** Immunohistochemistry markers utilized for B cell aggregate analysis (61–65).

**Antibody**	**Species**	**Source**	**Dilution**	**Target**
CD3 (SP7)	Rabbit IgG anti-mouse monoclonal	Abcam; ab16669	1:200	T cells
B220	Rat IgG anti-mouse	BD Biosciences; BDB557390	1:100	Mature B cells
IgG (subclasses 1, 2a, 2b, 3)	Goat anti mouse; 594 conj	Jackson Immunoresearch 715-585-150	1:200	Isotype-switched B cells
Laminin	Rabbit anti-mouse	Abcam; ab11575	1:500	Basement membrane
Ki-67	Rabbit anti-mouse	Abcam; ab15580	1:100	All active phases of the cell cycle
Active Caspase-3	Rabbit anti-mouse	Abcam; ab2302	1:100	Cell death
CD21/35	Rat anti-mouse	BD Biosciences; 553817	1:100	Follicular dendritic cells
GL7	Rat IgM anti-mouse; FITC conj	BD Biosciences; 562080	1:100	Germinal center B cells

All TMEV-IDD mice (*n* = 8) in our current immunofluorescence studies exhibited CNS inflammatory aggregates, albeit to varying degrees, with no detectable immune cell infiltration in sham-treated mice (*n* = 6).

### Statistical Analyses

Data generated in the present study were analyzed via Prism (version 6.0) software (GraphPad, San Diego CA). Data sets were assessed using a Pearson normality test to determine significant deviations from a normal distribution. Based on the normality results, the parametric Student's *t*-test or non-parametric Mann-Whitney U test was utilized to compare groups. The exact test used to perform analyses for each data set are denoted within the corresponding figure legend. In all cases, a *P* < 0.05 was considered significant.

## Results

### Isotype-Switched B Cells Predominate in the Spinal Cord and Associate With Intrathecal Antibody Synthesis During Chronic TMEV-IDD

**Figure 1:**
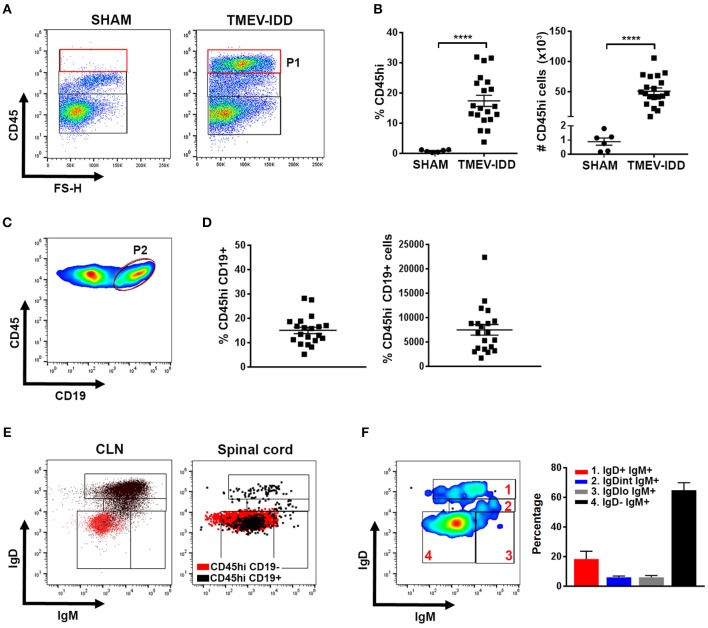
Isotype-switched B cells comprise the majority of spinal cord-infiltrating B cells during chronic TMEV-IDD. **(A)** Representative gating strategy for CD45^hi^ (P1) cells in the spinal cords of sham-treated or TMEV-infected mice at day 130 post-injection showing minimal CD45^hi^ cells in sham mice. **(B)** Scatter plots display CD45^hi^ percentage (left) and numbers (right) among total live cells in individual sham or TMEV-IDD mice with mean ± SEM for the group. **(C)** Representative gating strategy for CD45^hi^ CD19^+^ B cells (P2) and **(D)** scatter plots displaying CD19^+^ B cell percentages (left) and numbers (right) within CD45^hi^ cells for individual TMEV-IDD mice and mean ± SEM for the group. **(E)** Dot plots show IgD and IgM subpopulations in the CLN (left) and TMEV-IDD spinal cord (right). Gating strategy for B cell subpopulations was initially established in the CLN and then applied to spinal cord-infiltrating B cells. CD45^hi^ CD19^−^ non-B cell immune cells (red) were used as a negative control in both tissues. CD45^hi^ CD19^+^ B cells (black) were overlaid to confirm gating of IgD^−^IgM^−^ isotype-switched B cells. **(F)** Smoothed representative gating strategy (left) and mean percentages ± SEM **(**right) of B cell phenotypes among CD45^hi^ CD19^+^ spinal cord-infiltrating B cells identifying IgD and IgM subsets including (1) IgD^+^IgM^+^ naïve/early activated, (2) IgD^int^IgM^+^ activated, (3) IgD^−^IgM^+^ transitional/isotype-unswitched memory/ASC, and (4) IgD^−^IgM^−^ isotype-switched memory/ASC B cells. Data are representative of three independent experiments with individual TMEV-IDD (*n* = 6 to 8) or sham (*n* = 2) mice per experiment. Significant differences between sham and TMEV mice are indicated by ****(*p* < 0.0001) as determined by an unpaired Student's *t*-test.

To assess B cell phenotypes within the CNS of TMEV-IDD mice, flow cytometry was utilized to examine infiltrating B cells in spinal cords, i.e., a prominent site of inflammation, demyelination, and viral persistence during chronic TMEV infection (41, 46). CD45, a pan immune cell marker, was used to identify infiltrating immune cells in sham and TMEV-IDD spinal cords (Figure 1A; P1). Results revealed prominent CD45^hi^ immune cell infiltration in TMEV-IDD spinal cords (mean number = 50,841; SEM ± 5390), while sham-treated mice showed minimal presence of CD45^hi^-expressing cells (mean number = 887; SEM ± 257) (Figure 1B). Further analysis of infiltrating CD19^+^ B cells within total CD45^hi^ cells (Figures 1C,D; P2) revealed 15% (mean = 7,481 cells) were CD45^hi^CD19^+^ in TMEV-IDD spinal cords, while CD45^hi^CD19^+^ B cells were scarce in sham-treated mice (~15–20 cells; data not shown).

To define the differentiation state of spinal-cord infiltrating B cells, we identified naïve/early activated, activated, transitional or isotype-unswitched ASC/Bmem, isotype-switched ASC/Bmem utilizing IgD and IgM surface expression (Table 1; Figure 1E) (21, 23, 24). B cell immunophenotyping in chronic TMEV-IDD identified the majority of B cells (~60%) as being IgD^−^IgM^−^ isotype-switched ASC/Bmem phenotype (Figure 1F).

**Figure 2:**
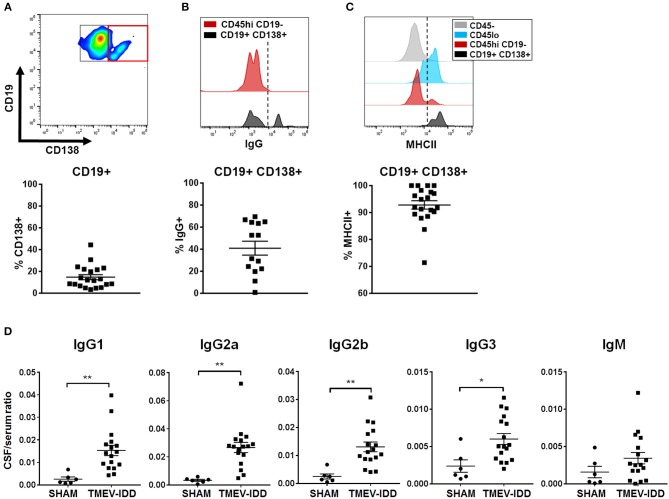
Accumulation of isotype-switched B cells, including ASC, coincides with intrathecal Ig synthesis. **(A)** Gating strategy for CD138^+^ ASC (red box) among CD45^hi^ CD19^+^ cells (top). Scatter plots showing total percentage of CD19^+^ CD138^+^ cells (bottom) among CD45^hi^ CD19^+^ B cells for individual TMEV-IDD spinal cords at day 130 p.i. with mean ± SEM for the group. **(B)** Histogram (top) depicts IgG expression among CD45^hi^ CD19^−^ non-B cell immune cells as a negative control (red) and IgG expression among CD19^+^ CD138^+^ ASC (black). Dotted line denotes cutoff for positive gating of IgG^+^ cells. Total percentage of IgG^+^ cells (bottom) among CD45^hi^ CD19^+^ CD138^+^ B cells for individual mice and mean ± SEM for the group. **(C)** Histogram (top) shows MHCII expression among CD45^−^ (astrocytes/neurons/oligodendrocytes; gray) as a negative control, with elevated expression among CD45^lo^ microglia (blue) and CD45^hi^ CD19^−^ non-B cell infiltrating immune cells (red) serving as positive controls. Histogram highlights elevated MHCII expression among CD19^+^ CD138^+^ cells (black). Dotted line denotes cutoff for positive gating of MHCII^+^ cells. Total percentage of MHCII^+^ (bottom) among CD45^hi^ CD19^+^ CD138^+^ B cells for individual mice and mean ± SEM for the group. **(D)** Immunoglobulin (Ig) protein levels were measured in individual CSF and serum samples using Luminex® technology to measure 6 Ig isotypes simultaneously. Scatter plots depict individual CSF/serum ratios of IgG1, IgG2a, IgG2b, IgG3, or IgM and mean ± SEM for the group. IgA showed no significant differences in CSF/serum ratio compared to sham. Significant differences between sham and TMEV mice are indicated by *(*p* < 0.05) or **(*p* < 0.01) as determined by an unpaired Student's *t*-test.

These data, combined with previous results showing pronounced ItAb in the CNS during chronic TMEV-IDD (45), led us to further characterize differentiated B cells accumulating during chronic disease. We examined the canonical surface marker of ASC, CD138 (Table 1). Initial analysis revealed that CD138^+^ cells expressed moderate levels of CD19 and 20% of total CD19^+^ cells expressed CD138 (Figure 2A). Among CD19^+^ CD138^+^ ASC, an average of 40% were class-switched IgG^+^ (Figure 2B) and the majority of CD19^+^ CD138^+^ ASC expressed MHCII (~90%; Figure 2C), suggesting a plasmablast phenotype.

Flow cytometric analysis revealed the majority of CNS-infiltrating B cells were comprised of an isotype-switched B cell phenotype and previous studies have documented elevated CSF Ab in TMEV-IDD (45). These findings led us to investigate if the presence of differentiated B cells associated with ItAb production in the CSF. Immunoglobulin protein levels were measured in the serum and CSF of sham-treated and chronic TMEV-IDD mice. IgG_1_ (*p* = 0.004), IgG_2a_ (*p* = 0.001), IgG_2b_ (*p* = 0.002), and IgG_3_ (*p* = 0.01) all displayed significantly elevated CSF/serum ratios during chronic TMEV-IDD (Figure 2D) relative to sham-treated mice. IgM CSF/serum ratio was also moderately elevated in TMEV-IDD compared to sham-treated mice but did not reach statistical significance. Elevated CSF/serum IgG isotype ratios indicated CSF IgG was intrathecally produced as BBB integrity is restored during early disease onset (41, 47), indicating that passive transfer of Ab from the serum was unlikely.

### Aggregation of Activated and Isotype-Switched B Cells Occurs in Meningeal and Perivascular Spaces

Diverse B cell differentiation phenotypes in TMEV-IDD spinal cords, including extensive isotype-switched ASC/Bmem accumulation and ItAb synthesis in chronic TMEV-IDD, provided evidence for CNS-compartmentalized B cell responses, a phenomenon suggested to occur during the chronic and progressive phases of MS (66).

**Figure 3:**
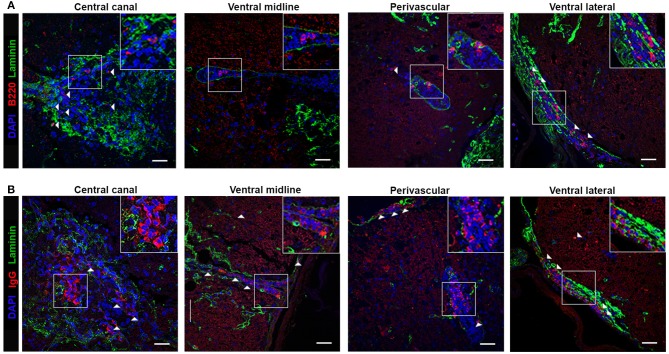
B cell localize to the meninges, perivascular, and parenchymal space in the Theiler's infected spinal cord. TMEV-infected decalcified spinal cords at day 130 p.i. were stained with DAPI (blue), laminin (green) and **(A)** B220 or **(B)** IgG (red) as indicated. Representative z stack projected compilations revealing B cell aggregation in the central canal, ventral midline, perivascular space, and ventral lateral meninges. White boxes indicate region selected for cropped insets in each image. White arrows highlight positive B220 or IgG staining as indicated. Z stack compilations represented 6-8 TMEV-infected mice. Scale bar = 50μm.

To determine B cell localization and possible ELF involvement in fostering immune responses during chronic TMEV-IDD, we examined B cells in spinal cords with intact meninges by immunofluorescent staining. We utilized the basement membrane component laminin to delineate vasculature and the meninges, common sites for immune cell aggregation in the CNS (18, 29, 36, 39). B cells were identified via B220, a surface marker present on immature and mature B cells but downregulated on terminally differentiated plasma cells, whereas IgG was utilized to identify isotype-switched B cells, including IgG expressing ASC and Bmem (Table 1). Analysis of B220^+^ (Figure 3A) or IgG^+^ (Figure 3B) cell localization relative to laminin in cervical and thoracic spinal cord sections showed prominent B cell infiltration in parenchymal, meningeal, and perivascular spaces. Both B220^+^ and IgG^+^ B cells accumulated proximal to the central canal, within the ventral midline vasculature, with evident perivascular cuffing, and frequent aggregation in the ventral lateral meninges, analogous to leptomeningeal immune cell aggregations present in MS patients.

### CNS Inflammatory Aggregates Accumulate in the Absence of Ectopic Lymphoid Follicle Markers

**Figure 4:**
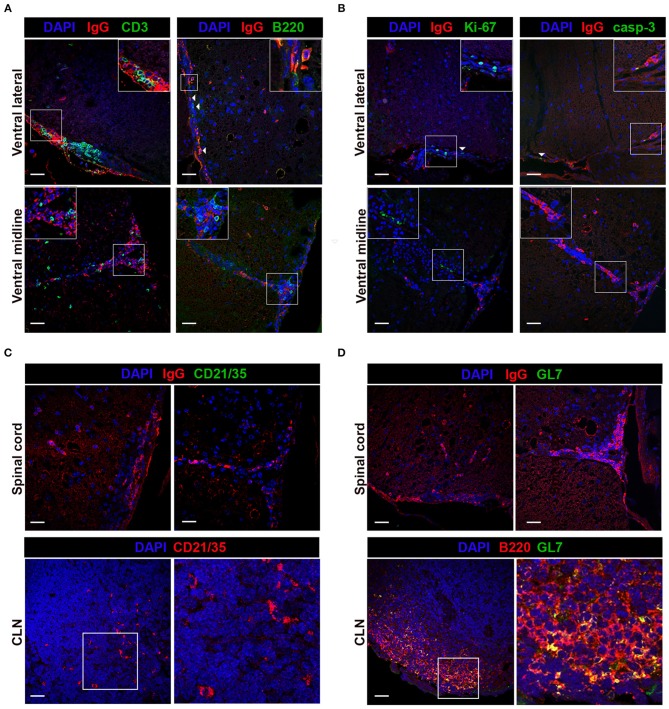
Immune cells aggregate in TMEV-IDD spinal cords in absence of conventional SLO-like ELF features. TMEV-infected decalcified spinal cords from day 130 p.i. were stained and assessed for markers of SLO-like ELF. Spinal cord representative images depict the ventral meninges and the ventral midline, regions with frequent cellular aggregation. White boxes indicate the region cropped for insets within each image. **(A)** CD3 or B220 (green), IgG (red), and DAPI (blue) were co-stained to assess co-aggregation and organization of infiltrating immune cells. Inset within IgG B220 costained representative shows B220^+^IgG^+^ double-positive B cell. **(B)** Ki-67 or caspase-3 (green), IgG (red) and DAPI (blue) were used to determine cell proliferation and cell death in B cell-containing aggregates. White arrows indicate presence of Ki-67 or caspase-3 immunoreactivity. **(C)** FDC presence in cellular aggregates was assessed in TMEV-IDD spinal cords using CD21/35 (green) with DAPI (blue) and IgG (red). As a positive control, CD21/35 immunostaining was confirmed at day 27 p.i. in CLN tissue using CD21/35 (red) and DAPI (blue). White box within CLN indicates the region cropped for the representative image (right) highlighting CD21/35^+^ cells. **(D)** GC B cells in TMEV-IDD spinal cords were identified using GL7 (green), with IgG (red), and DAPI (blue). CLN tissue (day 27 p.i.) was used to verify GL7 (green) immunostaining with B220 (red) and DAPI (blue). White box with CLN indicates the region cropped for the representative image (right) highlighting GL7^+^ B220^+^ (yellow) GC B cells. Representative z stack compilations from 6-8 mice. Scale bar = 50μm.

B220^+^ and IgG^+^ B cell accumulation within perivascular and meningeal regions led us to examine if these cellular aggregations displayed hallmark SLO-like ELF characteristics, including evidence for spatial organization, differentiation, and antigen-driven selection. Spatial organization was examined by identifying the localization of T cells, key immune cells involved in initiating and organizing ELF formation and in promoting antigen-driven B cell activation, differentiation, and survival (16, 35, 67). Immunofluorescent staining of CD3^+^ T cells and IgG^+^ B cells demonstrated T cells aggregated with isotype-switched IgG^+^ B cells in the ventral lateral meninges and ventral midline, although there was no evidence for a discrete organization of T and B cell regions, a characteristic feature of SLO and SLO-like ELF (Figure 4A). To evaluate B cell differentiation within the CNS compartment, we simultaneously assessed B220 and IgG B cell localization and aggregation within perivascular and meningeal cellular infiltrates. Co-staining of B220 and IgG within spinal cord sections revealed frequent localization of B220^+^, IgG^+^, and occasional B220^+^ IgG^+^ double-positive cells within cell aggregations in the ventral lateral meninges and ventral midline vasculature with minimal evidence of organized clusters containing IgG and B220-expressing B cells (Figure 4A).

Although cellular organization akin to SLO was not evident, simultaneous accumulation of T cells with immature, mature, and isotype-switched B cells in meningeal and perivascular spaces of TMEV-IDD spinal cords was apparent. To further evaluate whether immune cell aggregates expressed common features of SLO-like ELF, we utilized markers of proliferation and cell death, events indicative of ongoing antigen-driven selection within germinal center (GC) reactions in SLO and ELF (13, 16). Proliferation was assessed using Ki-67, and active caspase-3 was used as an indicator of apoptosis (Table 2). Immunohistochemical analysis of Ki-67 and caspase-3 expression revealed sparse immunoreactivity within meningeal and perivascular cellular aggregates containing IgG^+^ cells (Figure 4B). Further evaluation of perivascular and meningeal aggregates also showed the absence of the follicular dendritic cell (FDC) marker CD21/35, indicating an absence of FDCs, a specialized stromal cell in SLO and ELF involved in trapping and presenting antigen in B cell follicles and in secretion of B cell supporting chemokines, adhesion molecules, and trophic factors (Table 2; Figure 4C) (14). To evaluate GC formation, structures essential for antigen-driven selection, somatic hypermutation, and isotype switching, we utilized GL7, a surface antigen present at high levels on GC B cells during GC reactions in SLO and ELF (Table 2) (52, 68). Immunostaining of TMEV-IDD spinal cords showed no evidence of GL7 immunopositivity within immune cell aggregates containing IgG^+^ B cells (Figure 4D). Thus, histological assessment revealed minimal evidence for SLO-like ELF formation.

### CNS Infiltrating B Cells Display Elevations in Activation Markers During Chronic TMEV-IDD

**Figure 5:**
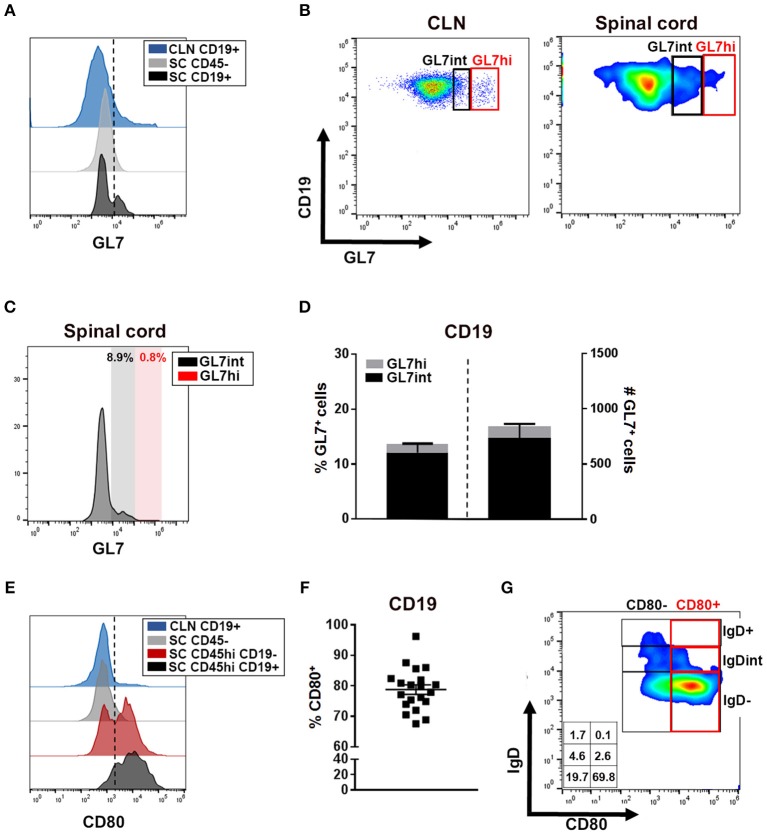
CNS-infiltrating B cells express markers of activation. **(A)** Histogram depicts GL7 expression on CLN CD19^+^ cells (blue) as a positive control, spinal cord CD45^−^ (astrocytes/neurons/oligodendrocytes; gray) as a negative control, and spinal cord-infiltrating CD19^+^ B cells (black). Dotted line delineates cutoff for positive gating of GL7. **(B)** Representative dot plot of GL7 expression with CLN (day 27 post infection -p.i.-) demarcating activated/pre-GC GL7^int^ (black box) and germinal center (GC) phenotype GL7^hi^ (red box) with the same gating applied to the representative smoothed dot plot of B cells isolated from the chronic TMEV-IDD spinal cord. **(C)** Histograms of the mean fluorescence intensity (MFI) of GL7 expressing cells among spinal cord CD45^hi^ CD19^+^ cells identifying minimal GL7^int^ (black) activated B cells and GL7^hi^ (red) GC phenotype B cells. **(D)** Stacked bar graphs display the mean ± SEM percentage (left bar and axis) and mean ± SEM number (right bar and axis) of GL7^int^ and GL7^hi^ B cells within total CD45^hi^ CD19^+^ GL7^+^ spinal cord infiltrating B cells. **(E)** Histogram depicts CD80 expression among CLN CD19^+^ B cells (blue) at day 27 p.i. showing restricted CD80^+^ cells, CD45^−^ (astrocyte/neurons/oligodendrocyte; gray) expression as a negative control, and CD45^hi^ CD19^−^ non-B cell immune cells (red) expression as a positive control. CD80 expression on CD45^hi^ CD19^+^ spinal cord infiltrating B cells is depicted in black. Dotted line delineates cutoff for gating CD80 positive cells. **(F)** CD45^hi^ CD19^+^ B cells and scatter plot showing individual percentages and group mean ± SEM of CD19^+^ CD80^+^ B cells within the spinal cord at day 130 p.i. **(G)** Representative dot plot showing expression of CD80^+^ predominately on IgD^−^ B cells in the spinal cord with corresponding percentages for each population indicated within the table inset. Results represent three independent experiments with 6 to 8 mice per experiment.

The absence of detectable GL7 expression in spinal cords by IHC was further confirmed by flow cytometry, a more sensitive method of detection. Flow cytometry showed minimal evidence for spinal cord-infiltrating B cells expressing a conventional GL7^hi^ GC phenotype as depicted in the TMEV-IDD CLN (Figures 5A,B; red box), though modest GL7 expression was observed on a small proportion of spinal cord-infiltrating B cells (Figures 5B,C). Among GL7-expressing B cells in the spinal cord, the mean fluorescence intensity (MFI) was comparable to GL7^int^ “activated” B cells (Table 1) in the CLN (Figures 5A,B) (23, 52, 68). GL7 expression analysis identified 10% or approximately 800 infiltrating B cells as expressing an “activated” GL7^int^ phenotype, with minimal detection of GL7^hi^ or “GC” phenotype infiltrating cells (Figure 5D). In TMEV-IDD spinal cords, GL7^int^ B cells were mostly IgD^−^, confirming that the GL7 expression was elevated on activated/transitional B cells (data not shown). Observed GL7^int^ expression among a proportion of B cells within the spinal cord implied these B cells had acquired an activated phenotype.

Following stimulation, including CD4 T cell-dependent help, GL7 surface expression is moderately upregulated, increasing B cell activation and functional capabilities, including antigen presentation (52, 53). To further assess B cell activation, we quantified the expression of CD80, a costimulatory molecule upregulated following stimulation by factors including BCR or CD40 engagement which, upon signaling, enhances B cell survival, proliferation, and differentiation (54, 69). Analysis of CD80 surface expression among CD45^hi^ CD19^+^ B cells revealed expression on approximately 80% of infiltrating B cells (Figures 5E,F). Among CD80^+^ B cells, the majority were an IgD^−^ phenotype, indicating CD80^+^ B cells include highly-activated, transitional, and/or Bmem phenotypes (Figure 5G). Extensive CD80 expression on CNS-infiltrating B cells suggested most B cells accumulating during chronic TMEV-IDD display an activated phenotype possibly driven by antigen and CD4 T cell-mediated help (54, 70, 71), or perhaps, by polyclonal activation (72).

### Elevated Transcript and Protein Levels of B Cell Trafficking Chemokines and Factors Supporting Differentiation and Survival

**Figure 6:**
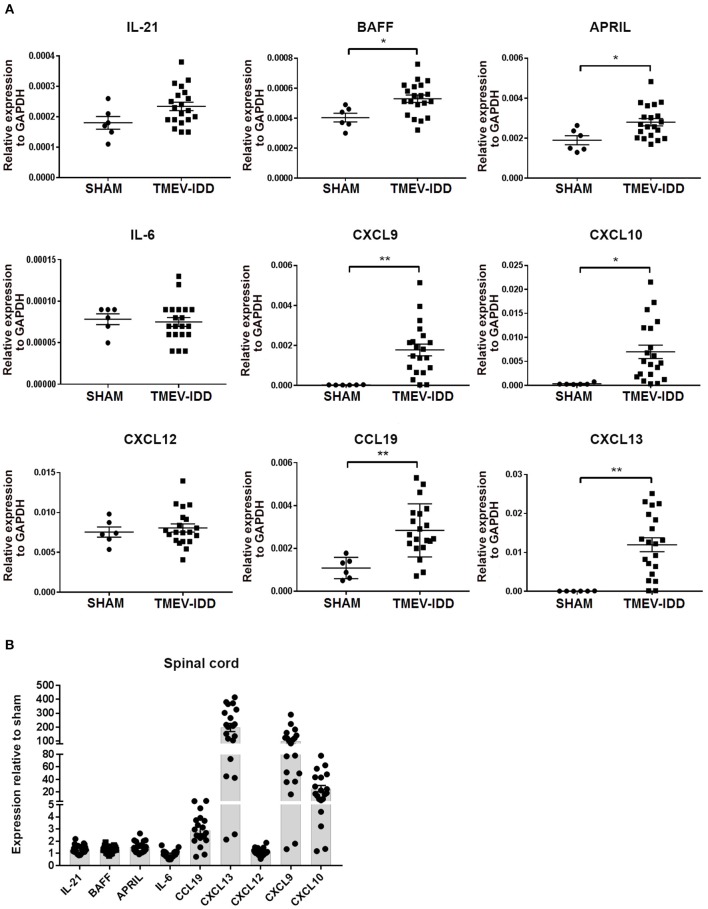
B cell accumulation in the central nervous system coincides with enhanced transcript expression of B-cell supportive chemokines. **(A)** Relative gene expression levels for IL-21, BAFF, APRIL, IL-6, CXCL9, CXCL10, CXCL12, CCL19, CXCL13 in the spinal cords of individual sham and chronic TMEV-infected mice (day 130 post-infection -p.i-), normalized by the housekeeping gene glyceraldehyde phosphate dehydrogenase (GAPDH) with mean ± SEM plotted for each group. **(B)** Scatter plot showing fold-changes in cytokine and chemokine expression compared to sham mice. The fold-change was obtained by normalizing the gene expression number to those of GAPDH, then comparing the samples to the average GAPDH-normalized gene expression in sham mice. Data depict the means ± SEM for at least 6 to 8 individual infected mice and 2 sham mice derived from three independent experiments. Significant differences between sham and infected are indicated (*P < 0.05; **P < 0.005). In all cases, a *P* value of <0.05 was considered significant, as determined by an unpaired Student's *t*-test or Mann-Whitney *U*-test.

Heterogeneous B cell differentiation phenotypes, including B cells displaying an activated phenotype, accumulating in parallel with intrathecal Ab synthesis led us to examine if cellular aggregations were still associated with factors involved in fostering B cell activation, survival, and accumulation in the absence of SLO-like ELF. Therefore, we evaluated whether factors promoting B cell trafficking, differentiation, and survival were present in chronic TMEV-IDD. IL-21 is a cytokine produced predominately by CD4 T cells and is essential for promoting B cell survival and differentiation (73, 74). Additionally, IL-6, BAFF, and APRIL are cytokines which play crucial roles in B cell survival and differentiation and are primarily produced by astrocytes within the CNS during viral infection and autoimmunity (19, 75–77). Gene expression analysis of these factors revealed constitutive expression of IL-21, IL-6, BAFF, and APRIL within spinal cords (Figure 6A), consistent with previous studies highlighting basal expression within CNS tissue (21, 25).

Further comparisons among sham-treated and TMEV-IDD mice showed modest upregulation of IL-21 (*p* = 0.06), with significant upregulation of APRIL (*p* = 0.01) and BAFF (*p* = 0.01) transcripts. IL-6 transcript levels were comparable to sham-treated mice (Figure 6A). Overall, IL-21, APRIL, and BAFF mRNA levels in TMEV-IDD spinal cords were twice as high as shams (Figure 6B).

CXCL9, CXCL10, CCL19, CXCL12, and CXCL13 are important for B-cell related trafficking and organization and are known to be produced in the CNS (21, 27, 32, 78). Analysis of chemokine transcript levels revealed constitutive expression of CCL19 and CXCL12 in spinal cords (Figure 6A), confirming previous results of basal expression in the CNS (21, 25). In TMEV-IDD mice, CXCL9 (*p* = 0.003), CXCL10 (*p* = 0.01), CCL19 (*p* = 0.002), and CXCL13 (*p* = 0.001) mRNA levels were all significantly increased compared to shams. Overall, CXCL13 showed a ~200-fold increase in expression, CXCL9 a ~100-fold increase in expression and CXCL10 a ~20-fold increase in expression, when TMEV-IDD spinal cords were compared to sham spinal cords (Figure 6B). Conversely, CXCL12 mRNA levels were similar in the spinal cords of TMEV-IDD and sham-treated mice.

**Figure 7:**
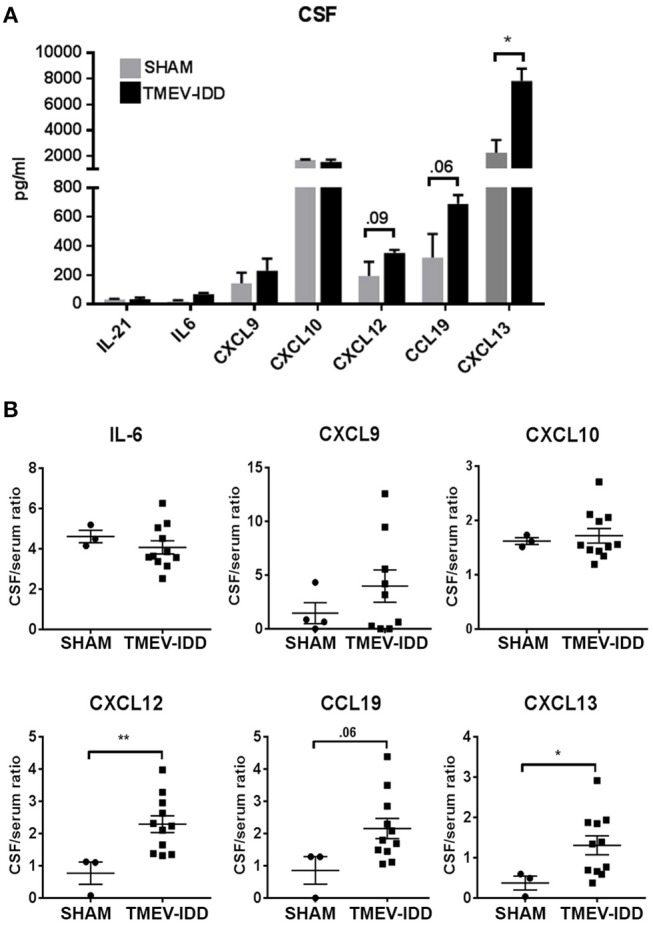
Selective B cell supportive chemokines are elevated in the cerebrospinal fluid during chronic TMEV-IDD. **(A)** Cerebrospinal fluid (CSF) levels (in pg/mL) and **(B)** CSF/serum ratios of B cell supporting cytokines in sham (*n* = 3–4) and chronic TMEV-infected mice (*n* = 11–12) at day 130 post-infection (p.i.) collected from three independent experiments. CSF levels are expressed as mean ± SEM for each group and CSF/serum ratios are expressed as dot plots showing individual values and the mean ± SEM for each group. Significant differences between sham and TMEV-infected mice are indicated (*P < 0.05; **P< 0.005) as determined by an unpaired Student's *t*-test.

To confirm that elevated transcript levels resulted in enhanced protein levels in the CNS compartment, we also evaluated protein levels in CSF from sham-treated and TMEV-IDD mice for all analytes previously tested by gene expression available for quantification by Luminex magnetic bead assay. Assessment of B cell differentiation and supporting factors, IL-21 and IL-6, confirmed previous gene expression results with minimal alterations in protein levels for both analytes in TMEV-IDD CSF samples compared to controls and overall low expression ranging from 10-30 pg/ml (Figure 7A). Further analysis of B cell trafficking and organizing chemokine protein levels revealed a modest increase in CXCL9 production in TMEV-IDD CSF (mean = 227 pg/ml) compared to sham (mean = 143 pg/ml), although results did not approach significance. Similarly, CXCL12 showed elevated production in TMEV-IDD (mean = 352 pg/ml) compared to sham (mean = 193 pg/ml) although results were only near to significance (*p* = 0.09). CCL19 and CXCL13 both displayed significantly increased expression, with CCL19 reaching an average of 689 pg/ml in TMEV-IDD CSF compared to sham levels of approximately 319 pg/ml (*p* = 0.02) and CXCL13 exhibiting the highest protein levels of CXCL13 at an average of 7820 pg/ml compared to sham levels of 2249 pg/ml (*p* = 0.01), coinciding with high upregulation in transcript levels detected by gene expression analysis in TMEV-IDD spinal cords. Conversely, CXCL10 protein levels in sham and TMEV-IDD spinal cords diverged from transcript levels, with comparable CXCL10 protein levels in sham and TMEV-IDD CSF (~1600 pg/mL), though CXCL10 protein levels in the CSF showed the second highest expression among all analytes assessed.

### B Cell Trafficking and Organizing Chemokines Are Intrathecally Produced During Chronic TMEV-IDD

The above data showing elevated transcript and protein levels of B cell trafficking and organizing chemokines led us to examine if these factors were also intrathecally produced. To assess if elevation in intrathecal production of B cell-related chemo-attractants and trophic factors was evident in the CSF of TMEV-IDD mice, we evaluated protein levels of B cell chemokines in the CSF and serum to establish a CSF/serum ratio. Results revealed moderately increased CXCL9 and CCL19 (*p* = 0.06) CSF/serum ratios, with significant increases in CSF/serum ratios among CXCL13 (*p* = 0.03) and CXCL12 (*p* = 0.005) in TMEV-IDD compared to sham-treated mice (Figure 7B). IL-6 and CXCL10 CSF/serum ratios revealed minimal alterations in chronic TMEV-IDD compared to sham-treated CSF samples. Elevated CSF/serum ratios in the absence of detectable increased BBB permeability [(47); manuscript submitted] suggest intrathecal production is responsible for the observed elevations in B cell trafficking chemokines in the CNS compartment.

## Discussion

Our experiments focused on elucidating B cell phenotypes and factors promoting persistent accumulation in the CNS compartment during chronic TMEV-IDD. Previous studies have shown a prominent involvement of the B cells in the CNS compartment during TMEV-IDD, including robust ItAb production and IgG^+^ and CD138^+^ B cells localizing to the CNS (47). Further studies highlighting upregulated B-cell immune response pathway genes in the deep CLN during the early phase of the disease and a relative downregulation during chronic TMEV-IDD also suggested a shift in B cell inflammation from the peripheral to CNS compartment during chronic TMEV-IDD (79). The spectrum of B cells involved in the CNS and their localization and organization within the CNS has not been extensively investigated and served as the foci of this work.

Our studies demonstrate that within the CNS during the progressive phase of TMEV-IDD, activated and isotype-switched B cells, including Bmem and ASC preferentially accumulate relative to other B cell phenotypes. These cells likely underlie the observed elevations in CSF Ab production, including ItAb of IgG1, IgG2a, IgG2b, IgG3. Present results revealed that the majority of CD138^+^ ASC express MHCII, indicating a plasmablast phenotype. Our data, as well as previous studies in TMEV-IDD (47), have shown that few CD138^+^ ASCs display Ki-67 immunopositivity, suggesting their proliferative capacity is decreased (47). It is possible that our flow cytometry results are skewed toward detecting a plasmablast phenotype B cell due to downregulation of CD19 on terminally differentiated plasma cells (80). However, other viral-induced encephalitic models such as MHV and Sindbis have confirmed high percentages of CNS-infiltrating CD138^+^ MHCII “plasmablast” phenotype B cells in the inflamed CNS (24). Since most classical plasmablasts continue to proliferate and thus are Ki-67^+^, the isotype-switched B cells exhibiting a CD19^hi^, CD138^+^, MHCII^+^ Ki-67^−^ phenotype in our experiments may represent a B cell phase intermediate between a plasmablast and terminally differentiated plasma cells, such as the early differentiated CD138^hi^ MHCII^+^ plasma cell phenotype previously documented in kidney inflammation and observed following sheep red blood cell immunization (80, 81). MHCII-expressing ASCs have essential implications during neuroinflammation as MHCII^+^ ASC can not only sustain Ab production but can also engage in antigen presentation to potentiate local immune responses.

B cell phenotyping in chronic TMEV-IDD spinal cords also highlighted extensive CD80^+^ B cell infiltration, suggesting the majority of accumulating B cells exhibit an activated phenotype associated with an enhanced capacity for antigen presentation, cytokine production, survival, and differentiation (54, 82). CD80 is also upregulated on Bmem (55, 56, 83). During chronic TMEV-IDD a high proportion of spinal cord-infiltrating B cells expressed an isotype-switched, differentiated phenotype (~70%), yet only 20% of total CD19^+^ were CD138^+^ ASC. These data, in combination with the observed high number of CD80^+^ B cells, suggest CD138^−^ isotype-switched B cells are Bmem in chronic TMEV-IDD. Future studies validating the presence of Bmem during chronic TMEV-IDD are required to assess conventional surface molecules present on murine Bmem, including co-expression of CD80 with PD-L2 and CD73 (84, 85). Investigating Bmem during chronic TMEV-IDD will be essential for our understanding of B cells in chronic demyelinating disease, including MS, as Bmem are antigen-experienced cells with potent antigen presentation capabilities, producing large amounts of pro- and anti-inflammatory cytokines, and have the potential to rapidly convert into antigen-specific ASC to sustain CNS-compartmentalized Ab synthesis (86, 87).

The maintenance of diverse B cell phenotypes within the CNS during neuroinflammatory diseases, including MS, is suggested to occur by two different mechanisms: (1) continuous recruitment from the peripheral compartment (88, 89) and (2) CNS compartmentalized inflammation, such as ELF formation (36, 37, 39, 90–92). In chronic and progressive MS, evidence for CNS compartmentalized B cell activity is supported by the relative absence of new gadolinium-enhancing lesions in the CNS caused by inflammatory cells derived from the periphery, and relatively reduced effectiveness for peripherally administered disease-modifying therapies, including anti-CD20, in eliminating CSF B cells (12, 93, 94). Furthermore, CNS compartmentalized inflammation including meningeal leukocyte aggregates, CSF B cells, and ItAb is associated with disease progression in MS (39). However, there is a significant gap in our understanding of the factors required for supporting B cell localization and functional activity within the CNS, including ELF formation, in MS and its animal models. During chronic TMEV-IDD as shown above, ItAb synthesis coincided with the accumulation of heterogeneous B cell subsets, including mature and isotype-switched B cells, frequently aggregating within meningeal and perivascular regions of the spinal cord, often localizing with T cells. Meningeal inflammatory aggregates lacked several conventional features of SLO-like ELF necessary for driving local B cell differentiation into antigen-specific Bmem or ASC. Spatial organization, heightened proliferation and cell death indicative of antigen-driven selection, FDC stromal networks, and detectable GL7 expression were all absent in chronic TMEV-IDD, a finding similar to previous studies in viral-induced demyelination models of MS and B cell-dependent EAE models (23, 29).

Use of the term ELF in the CNS encapsulates a diverse array of lymphoid tissue-like organization, and it remains to be determined if meningeal or perivascular aggregations can exhibit all the features of SLO-like ELF. The potential for the CNS to create an immune-competent environment resembling an SLO-like ELF requires a supportive stromal cell network to provide conventional trafficking and organizational chemokines such as CXCL12, CXCL13, CCL19/CCL21 and pro-inflammatory cytokines, including IL-17 and LTα, which can induce and perpetuate lymphocyte accumulation and organization (18). In our studies, conventional B cell trafficking, organizing, and trophic factors were expressed in the CNS and may aid heterogeneous B cell phenotype accumulation and ItAb synthesis. These common features of meningeal aggregations during neuroinflammation occurred in the absence of SLO-like ELF formation and GC markers essential for fostering antigen-driven selection of immune cells and inducing somatic hypermutation, class switch recombination, and local differentiation. Specifically, discretely organized T and B cell follicles, supportive FDC stromal cells, Ki-67^+^ proliferating and caspase-3^+^ cells indicating ongoing antigen-driven selection, and germinal center GL7^hi^ B cells were searched for, but not found, in our studies in the chronic phase of TMEV-IDD.

The characteristics of the meningeal and perivascular clusters of inflammatory cells in TMEV-IDD may present insights into the controversial issues surrounding the existence and role of ELFs in MS. Patients with RRMS and progressive forms of MS exhibit meningeal inflammatory aggregates (18, 36, 37, 39, 95–99), yet the presence of leukocyte aggregations does not confirm the meninges provide a niche for SLO-like ELF formation. Moreover, the pathology and molecules associated with meningeal inflammation have not been definitively determined. For instance, in MS there is no evidence thus far that the leukocyte aggregations in the CNS involve somatic hypermutation, including activation-induced deaminase (AID) expression, an enzyme essential for hypermutation of variable Ig genes and increased B cell receptor affinity for the antigen. In the viral-induced mouse model of demyelination, MHV, CNS inflammatory aggregates were observed yet AID transcript expression was absent among sorted B cells isolated from the CNS (21). Thus, leukocyte aggregations described in the CNS in MS may more closely resemble the “immature,” non-SLO-like ELFs described in our experiments in TMEV-IDD, in contrast to ELFs outside of the CNS in chronic inflammatory diseases which are more SLO-like such as inducible forms of lymphoid tissue, i.e., iBALT (13, 16). This difference between CNS lymphoid aggregates and ELFs in inflammatory conditions outside of the CNS may be due to an inherent inability of the CNS to develop and organize SLO-like ELFs, even with chronic inflammation.

Our studies did not document SLO-like ELF in driving B cell persistence in the CNS compartment but revealed frequent immune cell aggregation within meningeal spaces. Moreover, upregulation of markers, including GL7 and CD80, on B cells in chronic TMEV-IDD suggests the majority are highly activated and may be antigen-experienced (52–54, 68, 69). It remains unclear whether B cells acquire an activated phenotype in the peripheral or CNS compartment or the exact mechanisms driving activation (polyclonal activators vs. CD4 T cell help). Frequent immune cell aggregation in both meningeal and perivascular spaces and CNS-infiltrating B cells displaying an activated/helped phenotype suggest CD4 T cell-dependent activation may occur in the CNS compartment. The meninges are known to be a niche for harboring cells essential for promoting ongoing T cell activation and effector responses, suggesting B cell activation may be supported by similar mechanisms (100, 101). The abundance of T cells in meningeal and perivascular aggregates in the CNS during chronic TMEV-IDD resembles immune aggregates described in other viral infections of the CNS as well as in SPMS patients (23, 29, 37). Although studies examining B and T cell contact-dependent interactions within the CNS remain limited, following MHV infection, B and T cell interactions were evident within meningeal spaces in the brain which may promote local B cell activation (23). It remains to be determined if CD4 T cells are necessary and/or sufficient for propagating B cell activation, sustained accumulation, and potential differentiation in the CNS in mouse models of MS and MS patients. An important factor derived from T cells which supports B cell survival and differentiation in SLO, IL-21, showed minimal elevations during chronic TMEV-IDD in contrast to reports of elevated IL-21 production in the CNS during the acute phase of viral-induced encephalitis (21, 25). Nonetheless, increased GL7 and CD80 expression suggests most infiltrating B cells during chronic TMEV-IDD exhibited a contact-dependent CD4 T cell “helped” phenotype. Further studies are needed to determine if T cell secreted factors or contact-dependent mechanisms are essential for sustaining B cell accumulation and activation within the CNS compartment.

Our results and previous studies (19–21, 25, 27, 28) highlight that diverse factors may foster persistent B cell accumulation and ItAb in the CNS compartment. These factors may range in complexity from the expression of adhesion molecules such as CEACAM1, which alone can promote meningeal aggregation, to ectopic expression of lymphoid chemokines, cytokines and trophic factors supporting aggregation, to well-defined ELF resembling SLO (16, 18, 35, 102). Our study demonstrates that fully developed ectopic germinal centers are not required to support diverse B cell phenotypes and chronic CNS ItAb production. Irrespective of the level of organization, meningeal aggregates have been extensively associated with underlying cortical demyelination, gray matter pathology, diffuse axonal and neuronal loss (95–99, 103, 104). Thus, it is imperative to further delineate factors supporting meningeal aggregations, as potential mechanisms for dissociating pathogenic inflammatory aggregations in MS and other neuroinflammatory diseases will likely depend on the level of organization and molecular/cellular factors sustaining these structures. A limitation of the current study characterizing CNS compartmentalized inflammation in a chronic model of MS is the absence of mechanistic studies linking inflammatory aggregates with disability and/or neurodegeneration. Nonetheless, our data provide an important platform for investigating the role of inflammatory aggregates in neurodegeneration and for probing targets to disturb aggregates. Previous studies documenting neuronal death, axonal damage, and demyelination within the ventral regions of the spinal cord (40, 46, 105–107) in concert with our findings of frequent inflammatory aggregation in the ventral spinal cord, suggest a possible role of inflammatory aggregates in adjacent neurodegeneration.

Overall, the data show the ability for the CNS to foster isotype-switched B cell accumulation, ItAb synthesis, and production of B cell related chemokines and trophic factors, and meningeal inflammatory aggregates within the CNS compartment during a chronic, progressive mouse model of MS.

## Data Availability

All raw data and protocols used to generate datasets for this study will be made available by the authors upon request to any qualified researcher.

## Ethics Statement

This study was carried out in accordance with the recommendations of the Institutional Animal Care and Use Committee. The protocol was approved by the Institutional Animal Care and Use Committee.

## Author Contributions

KD designed the experiments and conceived the original idea with the supervision of AP and FG. KD and DR carried out all experiments. KD conducted all data analysis and data interpretation. KD wrote the manuscript with support from AP and FG.

### Conflict of Interest Statement

The authors declare that the research was conducted in the absence of any commercial or financial relationships that could be construed as a potential conflict of interest.
